# MACC1 Promotes the Progression and Is a Novel Biomarker for Predicting Immunotherapy Response in Colorectal Cancer

**DOI:** 10.1155/2022/8326940

**Published:** 2022-07-14

**Authors:** Man Xiong, Mingsen Wang, Youhong Yan, Xiaowu Chen, Wanwei Guo, Ming Xu, Shaoyan Guo, Yeyang Wang

**Affiliations:** ^1^School of Nursing, Guangzhou University of Chinese Medicine, Guangzhou 510006, Guangdong, China; ^2^Department of Gastroenterology, Guangdong Second Provincial General Hospital, Guangzhou 510030, Guangdong, China; ^3^Department of Orthopedic, Traditional Chinese Medicine Hospital, Puning, Jieyang 515300, China; ^4^Department of Spine Surgery, Guangdong Second Provincial General Hospital, Guangzhou 510030, Guangdong, China

## Abstract

**Aims:**

As one of the most prevalent malignant diseases in the world, the mechanisms of metastasis in colon cancer are poorly understood. The aim of this study was to investigate the role of the HGF/c-MET axis in the proliferation and metastasis in colon cancer.

**Methods:**

The effect of MACC1 on cell proliferation and metastasis was analyzed through a series of *in vitro* experiments. The role of MACC1 in cancer cells was demonstrated by overexpression and silencing of MACC1 in gain or loss function experiments. To investigate the relationship between MACC1 and c-MET/HGF, we detected c-MET protein expression by disrupting with or overexpressing MACC1. The bioinformatics analysis was used to investigate the correlation between MACC1 and c-MET, and the c-MET expression after the interference of HGF with MACC1 was determined. Subsequently, the function of c-MET was verified in colon cancer cells by a series of experiments. The mouse tumor transplantation model experiment is most suitable *in vivo*.

**Results:**

The results indicated that the overexpression of MACC1 could accelerate proliferation and facilitate metastasis in colon cancer cell lines. Furthermore, c-MET was determined to be the downstream regulator of MACC1. The addition of HGF could stimulate the expression of MACC1. With further exploration, we proved that c-MET is downstream of MACC1 in colon cancer and that overexpression of c-MET in colon cancer enhances cell proliferation and migration capability. At last, MACC1 expression level negatively correlates with the infiltration levels and several immune checkpoint biomarkers. High MACC1 expression has a lower response rate with ICIs in COAD.

**Conclusions:**

We found that, under the regulation of the MACC1/HGF/c-MET axis, the proliferation and metastasis of colorectal cancer are increased by MACC1, which can be a novel biomarker for predicting ICIs response in colorectal cancer. Our findings provide a new idea for the targeted treatment of colorectal cancer.

## 1. Introduction

As one of the most common malignant tumors of the digestive system, the incidence of colon cancer is increasing year by year and has surpassed the incidence of cardiovascular and cerebrovascular diseases [1]. It seriously threatens human health both physically and mentally. While the early identification and treatment of colon cancer have made great progress, the recurrence and metastasis of the tumor still severely impede the prognosis [2]. Hence, how to inhibit the recurrence and metastasis of colon cancer has become a key avenue for research worldwide. Currently, there are several targets for clinically developed HGF/MET signaling pathway [3–5]. However, for a number of unclear reasons, most of the HGF/c-MET targeted drugs have been ineffective or discontinued in clinical trials. Therefore, developing other drug targets will be a new way to develop targeted treatment for treating colon cancer.

Using genome-wide searches, Stein et al. identified metastasis-associated in colon cancer-1 (MACC1) as an important promoter of metastasis in human colon cancer [6]. MACC1, an oncogene on human chromosome 7p21.1, is an important premetastasis factor of human colon cancer. In recent years, more and more pieces of evidence have demonstrated that MACC1 plays a role in the occurrence and progression of tumors [7, 8]. MACC1 has been found to be less expressed in normal tissues but more overexpressed in colon, gastric, lung, esophageal, and other peritoneal tumor tissues [9–12]. Previous studies demonstrated that MACC1 can regulate the expression of the oncogene c-MET and promote epithelial-mesenchymal transformation, migration, proliferation, angiogenesis, and drug resistance in various tumor cells through the HGF/c-MET/MAPK and HGF/c-MET/AKT pathways [13–15]. MACC1 can activate hepatocyte growth factor (HGF), and the activated HGF can easily bind to c-MET, which promote the phosphorylation of c-MET, thereby inducing the proliferation and invasion of gastric cancer [16–19].

HGF is the natural ligand of c-MET, and thus c-MET is also known as the hepatocyte growth factor receptor (HGFR) [20]. Binding to HGF leads to dimerization and activation of c-MET receptors, which promotes the proliferation, migration, and invasion of tumor cells. c-MET can be activated by several abnormal factors and is involved in the occurrence and progression of different malignant tumors; thus, it is the “driving factor” of tumor occurrence [21, 22]. In preclinical studies, abnormal activation of the c-MET signaling pathway has been observed to be associated with poor clinical outcomes [23].

In our study, we found that MACC1 has significantly higher expression in colon cancer tissues than in the surrounding colon tissues. There is a positive correlation between the expression of MACC1 and the proliferation and migration of colon cancer cells. Ablation of MACC1 downregulates the expression of Ki67, MCM, and N-cadherin, thereby inhibiting cell migration and invasion. Meanwhile, the expression of c-MET in colon cancer cells varied with the change of MACC1 and showed positivity for colon cancer, and c-MET promoted the development and progression of colon cancer. Finally, in the mouse model, the effect of MACC1 on colon cancer was consistent with that in the cellular model. The exploration of the MACC1/HGF/c-MET pathway in colon cancer provides favorable evidence for drug targeting of MACC1 and regulation of MACC1/HGF/c-MET in colon cancer therapy.

## 2. Materials and Methods

### 2.1. Collection of Data

All TCGA expression data sets for analysis were downloaded from an online database. The original reading count was extracted from the file with the suffix “htseq. counts.” A limited number of cancer samples and cancer datasets from normal samples were used to accurately identify differentially expressed genes. Normal samples were defined as those labeled “solid tissue normal,” and the analysis did not include any normal samples. Pan-cancer at https://starbase.sysu.edu.cn/ analyzed the survival curves of 135 low-expression MACC1 cases and 135 high-expression MACC1 cases, and the expression of MACC1 in 471 colon cancer adenocarcinoma tissues and 41 normal tissues was also analyzed. We called the relationship between MACC1 and MET in linked omics (https://www.linkedomics.org/admin.php).

### 2.2. Patient Samples and Cell Culture

As approved by the appropriate ethics committee, freshly colon cancer tissues (12 pairs) were obtained. Human colon cancer cell lines, including HT-29, SW620, HCT116, and SW480, and normal oral epithelium cell line (NCM460) were obtained from the Shanghai Cell Bank of the Chinese Academy of Science (Shanghai, China). The cells were cultured in RPMI-1640 (Gibco, CA, USA) containing 5% CO_2_ at 37°C. The culture medium was 10% FBS and 1% Penicillin-Streptomycin Solution, which were both from Gibco, CA, USA.

### 2.3. Cell Transfection

According to the manufacturer's protocol, we transfected SW480 and HCT-116 cells with 2 *μ*g pcDNA3.1-MACC1 (referred to as MACC1 plasmids), 2 *μ*g control pcDNA3.1 plasmids (referred to as control plasmids), pcDNA3.1-c-MET (referred to as c-MET plasmids), and 2 *μ*g control pcDNA3.1 plasmids (referred to as control plasmids) for 48 hours. All the materials were purchased from Shanghai GenePharma Co., Ltd. The transfection was conducted using Lipofectamine® 2000 reagent (Invitrogen, Thermo Fisher Scientific, Inc.). The efficiency of transfection was measured by reverse transcription-quantitative (RT-q) PCR 48 hours after transfection. In the test, the untreated cells were used as controls.

### 2.4. Cell Viability Assay

In order to analyze cell viability, SW480 and HCT-116 cells were inoculated in 96-well plates with a 100 *μ*L culture medium in each well, and the density of cells was 5 × 10^3^ cells per well. Cells were inoculated in triplicate. We used 3-(4,5-dimethylthiazol-2-yl)-2,5-diphenyl tetrazolium bromide (MTT) assay (Sigma, USA) to measure the cell proliferation 0, 24, 48, and 72 hours after transfection.

### 2.5. EdU Proliferation Assay

To assess cell proliferation, SW480 and HCT-116 cells were incubated in 96-well plates under standard conditions in complete media and transfected the next day. Cell proliferation was measured using the incorporation of 5-ethynyl-2′-deoxyuridine (EdU) 48 hours after transfection using the EdU Cell Proliferation Assay Kit (Ribobio, Guangzhou, China). The cells were incubated with 50 *μ*M EdU for 6 hours, followed by fixation and permeabilization, and stained with EdU. The cell nuclei were stained with 1 *μ*g/ml DAPI (Sigma) for 20 minutes. Finally, fluorescence microscopy was used to determine the proportion of the cells incorporated in EdU.

### 2.6. Colony Formation Assays

First, we added 2 mL of medium, which contained 10% FBS, to each well of 6-well plates of cells (0.7 × 10^3^ per group). The 6-well plates were incubated in a humidified atmosphere containing 5% CO_2_ at 37°C for 14 days. During the incubation, the medium was changed every three days. Then, the cell surfaces were washed with PBS, fixed with 4% paraformaldehyde for 10 minutes, stained with Giemsa (Sigma, USA) for 50 minutes, and rinsed with PBS. Counts > colonies 50 mm in diameter.

### 2.7. RT-qPCR

We used Maxima SYBR Green/ROX qPCR Master Mix (Thermo Fisher Scientific, Waltham, MA, USA) for the RT-qPCR analysis. Then, total RNA was extracted from SW480 and HCT-116 cells in TRIzol® reagent (Invitrogen, Carlsbad, CA, USA). We calculated the 260/280 nm absorbance ratio to evaluate the quality of RNA and used a microplate reader to quantify the concentration. RT-PCR was conducted with 100 ng total RNA. Finally, the relative mRNA expression level was calculated by the 2CT method. The prim sequences are shown in Table 1.

### 2.8. Western Blot Assay

We homogenized colon cancer tissues with a Beads crusher and lysed the homogenized tissues and the surrounding adherent cells with RIPA buffer (08714-04, Nacalai Tesque). Then, we separated 120 *μ*g proteins using SDS-PAGE and transferred them to polyvinylidene difluoride membranes. Subsequently, we washed and blocked the membranes and incubated them with the specific MACC1 primary antibody (ab226803, dilution 1 : 1000), Ki-67 (ab92742, 1 : 1000), PCNA (ab92552, dilution 1 : 1000), vimentin (ab92547, dilution 1 : 2500), E-cadherin (ab76319, dilution 1 : 5000), N-cadherin (ab76011, dilution 1 : 6000), MCM (ab108935, dilution 1 : 1000), and GAPDH (#4970, dilution 1 : 2000). The obtained samples were then incubated with horseradish peroxidase-conjugated secondary antibodies (ab6789 or ab6721, dilution 1 : 1000). All materials were purchased from Abcam, Cambridge, MA, USA, except for GAPDH, which was obtained from Cell Signaling Technology, Inc. and Danvers, CO, USA. Finally, an enhanced chemiluminescence assay kit was used to visualize immunocomplexes (ECL Plus Western Blotting Detection Reagents, GE Healthcare, Buckinghamshire, UK).

### 2.9. Wound Healing Assay

Cells were grown to 70–80% confluence. Scratches were introduced into the cell monolayer, and then the cells were cultured in DMEM. We measured the migration distance at 0 hours and 12 hours after scratching.

### 2.10. Transwell Migration Assay

We collected 1 × 10^4^ SW480 and HCT-116 cells in a 150 *μ*l serum-free medium and spread them onto the upper chamber of a transwell plate. The lower chamber was filled with 700 *μ*l of the medium, which contained 10% FBS. The plates were incubated for 24 hours at 37°C. Then, we fixed the membranes in cold methanol and stained them with crystal violet.

### 2.11. SiRNA Interference Assay

GenePharma (Shanghai, China) synthesized three siRNA sequences that targeted different sites of human MACC1 and c-MET mRNA. Table 1 lists the synthesized siRNA sequences. In the tests, scrambled siRNAs were used as negative controls. We selected the sequence with the best interfering effect for further study.

### 2.12. *In Vivo* Study

Ten 5-week-old male BALB/C nude mice with a weight in the range of 17 to 20 g were purchased from the Guangdong Medical Laboratory Animal Center. All the nude mice were placed in an environment with a cycle of 12-hour light and 12-hour dark. The humidity was maintained in the range of 50% to 60%, and the temperature was kept in the range of 22°C and 24°C. The study mice can freely access water and food. All the nude mice were given one week to adapt to the environment. According to their body weight, the mice were randomly divided into two test groups, that is, the vector group and the MACC1 OE group, and each group contained 5 nude mice. Mice were subcutaneously inoculated with 2 × 10^6^ cells on the right side. The health and behavior of the animals were monitored every other day, the tumor growth was measured every 7 days, and the tumor was finally removed as planned. Tumor was collected at 30 days to measure volume and weight. The mice were anesthetized with 1% pentobarbital sodium *via* intraperitoneal injection and euthanized by cervical dislocation.

### 2.13. TISIDB and Immunophenoscore Analysis

The correlation between MACC1 and immunomodulators and tumor-associated immune cells was analyzed by TISIDB (https://cis.hku.hk/TISIDB/) [24].

The four main types of genes identified for immunogenicity were used to calculate immunophenoscore (IPS), which was used to predict the correlation between MACC1 expression and patients' response to Immune checkpoint inhibitors (ICIs) in colon adenocarcinoma (COAD). A higher IPS score indicated higher immunogenicity and a stronger response to ICIs. IPSs of TCGA COAD patients were downloaded from The Cancer Immunome Atlas (TCIA) [25].

## 3. Results

### 3.1. MACC1 Is Overexpressed in Colon Cancer Tissues and Cell Lines

**Figure 1:**
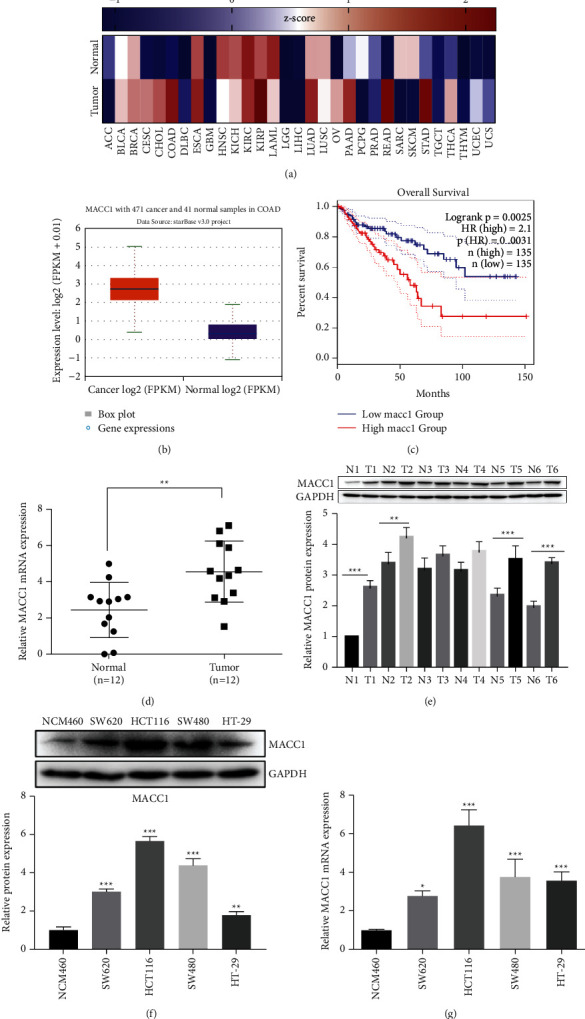
MACC1 expression in normal colon tissue and colon cancer. (a) Heatmaps of MACC1 expression in various TCGA tumors and normal tissues. (b) MACC1 with 471cancer and 41 normal samples. (d) The mRNA levels of MACC1 in collected tumor tissues and surrounding noncancerous tissues (*n* = 12). (e) MACC1 protein levels in tumor tissues and surrounding noncancerous tissues were determined by the western blot quantitative method (*n* = 12). (f) The expression and statistics of MACC1 in normal and colon cancer cells by western blot. (g) The expression of MACC1 in normal colon and colon cancer cells was detected by RT-qPCR. The results are shown as means ± SEM; ^*∗*^*P* < 0.05, ^*∗∗*^*P* < 0.01, and ^*∗∗*^*P* < 0.001.

To investigate the effect of MACC1 on colon cancer, we extracted the expression pattern of MACC1 in various cancers in the TCGA database [26]. Notably, the expression of MACC1 was upregulated in multiple mucosa malignancies, including colon adenocarcinoma (colon cancer), rectal carcinoma (READ), and stomach adenocarcinoma (STAD). Apparently, MACC1 is closely associated with digestive cancers (*P* < 0.01, Figure 1(a)). We studied the role of MACC1 in COAD and extracted the expression levels of MACC1 in colon cancer tissues and normal tissues from the TCGA database (*P* < 0.01, Figure 1(b)). Tissue microarrays containing 270 colon cancers in two groups, that is, MACC1^high^ and MACC1^low^, were used to analyze the prognostic value of MACC1 in colon cancer. It was obvious that MACC1^high^ patients presented a favorable prognosis (*P* < 0.01, Figure 1(c)). In order to validate the data from the online database, we analyzed the expression level of MACC1 in 12 pairs of colon cancer tumor tissues and surrounding nontumor tissues using RT-qPCR. From the result, MACC1 in colon cancer tissues had significantly upregulated expression (*P* < 0.01, Figure 1(d)). We randomly selected 6 pairs of carcinomatous and paracancerous tissue running proteins, and the MACC1 in carcinomatous tissue was higher than that in paracancerous tissue, which was consistent with the results of RT-qPCR (*P* < 0.01, Figure 1(e)). In addition, the hazard ratio of MACC1 (HR = 2.1, *P*=0.0031) was also calculated, and the result suggested that MACC1 is a prognostic factor for colon cancer. Furthermore, the mRNA expression level of MACC1 in multiple colon cancer cell lines (SW480, HCT116, SW620, and HT-29) and one normal colon epithelium cell line (NCM460) was examined. The result indicated that the expression of MACC1 in colon cancer cell lines was also markedly higher (*P* < 0.01, Figure 1(f)). The same conclusion was also confirmed by the protein level detected with western blot (*P* < 0.01, Figure 1(g)).

### 3.2. MACC1 Promotes Cell Proliferation and Metastasis in Colon Cancer

**Figure 2:**
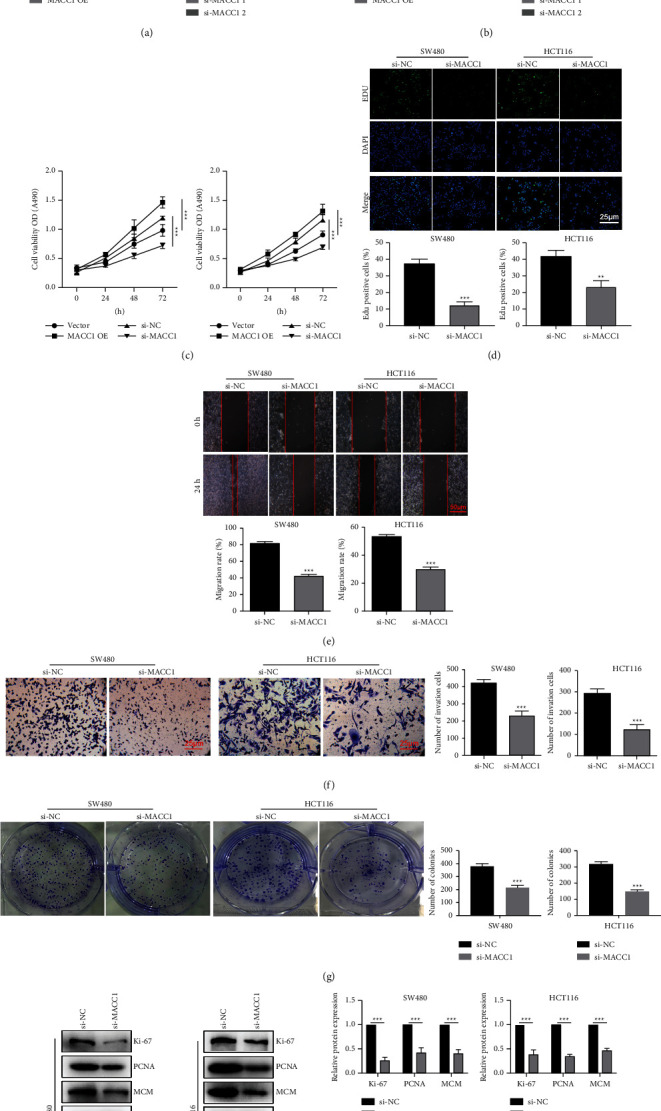
The expression of MACC1 in SW480 and HCT116 cell lines. (a) RT-qPCR validated the MACC1 overexpression and interference efficiency. (b) Cell viability was determined by CCK-8 assay after the overexpression of or interfered with MACC1. (c) Western blot validated the MACC1 overexpression and interference efficiency. (d) EdU detected proliferation after interference with MACC1. (e) Cell migration was determined by a scratch assay after MACC1 interference. (f) Cell invasion was detected after interference by the MACC1 invasion assay. (g) Clone formation assay validated the interference efficiency of MACC1. (h) Cell proliferation markers and statistics were measured by WB after interference with MACC1. The results are shown as means ± SEM; ^*∗*^*P* < 0.05, ^*∗∗*^*P* < 0.01, and ^*∗∗*^*P* < 0.001.

In order to further explore the regulatory role of MACC1 in colon cancer, we conducted a series of experiments on colon cancer cell lines related to malignant tumors. MACC1 expression in colon cancer cell lines was compared with the baseline expression levels. We constructed cell lines where HCT116 and SW480 overexpressed MACC1, and SW480 and HCT116 constructed cell lines with MACC1 knockdown. Cells overexpressing MACC1/knockout were constructed by lentivirus and siRNA, respectively. Overexpression and knockout efficiency were detected by RT-qPCR and western blot. The expression of MACC1 in the constructed cell lines had significantly up/downregulation (*P* < 0.01, Figure 2(a)). Furthermore, proliferation-related proteins, Ki-67/PCNA/MCM, were upregulated in cells overexpressing MACC1 while downregulated in cells with MACC1 knockdown (*P* < 0.01, Figure 2(b)). The MTT assay was used to assess the impact of MACC1 expression on the proliferation of colon cancer cells. After 72 h of observation, overexpression of MACC1 led to a significant increase in the proliferation of colon cancer cells. In contrast, the downregulation of MACC1 achieved the opposite effect (*P* < 0.01, Figure 2(c)). In the EdU cell proliferation assay, MACC1 overexpressing colon cancer cells had much stronger staining intensity, while MACC1 knockdown colon cancer cells had significantly weaker staining intensity, demonstrating the positive effect of MACC1 on the growth of colon cancer (*P* < 0.01, Figure 2(d)). Colon cancer is a highly metastatic form of cancer. In order to assess the influence of regulation of MACC1 on the proliferation, migration, and invasion of colon cancer cells, we conducted a cloning experiment, wound healing, and invasion experiment. The results showed that the overexpression of MACC1 accelerated the proliferation, migration, and invasion rate of colon cancer cells (*P* < 0.01). In contrast, the downregulation of MACC1 reduced the proliferation, migration, and invasion rates of colon cancer cells (*P* < 0.01, Figures 2(e)–2(g)). Subsequently, we intervened with MACC1 and collected cells to detect key proteins related to the ability of proliferation and EMT in cells. The results indicated that MACC1 knockout inhibited the ability of proliferation and EMT of colon cancer cells (*P* < 0.01, Figure 2(h)).

### 3.3. The Expression of c-MET Is Positively Regulated by MACC1

**Figure 3:**
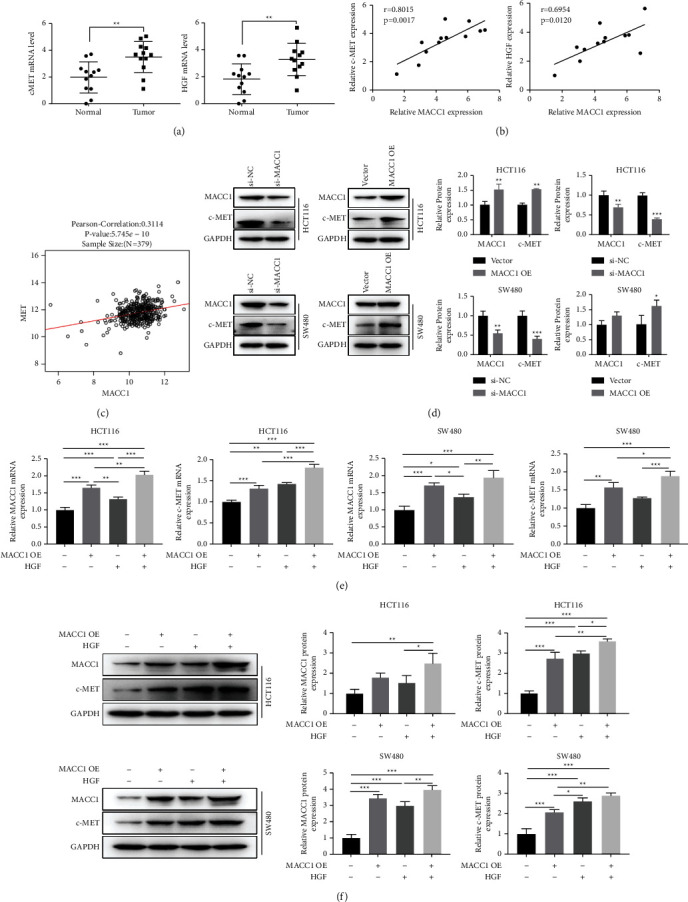
MACC1 regulates HGF/c-MET in HCT116 cells. (a) The mRNA levels of c-MET (left) and HGF (right) in tumor tissues and the surrounding noncancerous tissues (*n* = 12). (b) The mRNA levels of MACC1, c-MET, and HGF in tumor tissues (*n* = 12). (c) The analysis of the correlation between MACC1 and c-MET. (d) c-MET expression was determined by WB after interference and overexpression of MACC1. (e) QPCR examined interference and overexpression of MACC1 and MACC1 and HGF for the induction of c-MET expression in cells. (f) Statistical plot of c-MET expression detected by WB after interference and overexpression of MACC1. The results are shown as means ± SEM; ^*∗*^*P* < 0.05, ^*∗∗*^*P* < 0.01, and ^*∗∗*^*P* < 0.001.

We detected the expressions of c-MET and HGF in the tumor tissues and paracancerous tissues and found much higher expressions of c-MET and HGF in tumor tissues than in the paracancerous tissues (*P* < 0.01, Figure 3(a)). In the collected tissues, MACC1 and c-MET, MACC1, and HGF had a linear relationship (*P* < 0.01, Figure 3(b)). In GSEA data analysis, MACC1 and c-MET were positively correlated to a certain degree (*P* < 0.01, Figure 3(c)). Previous studies have reported that the expression of c-MET was possibly under the regulation of MACC1 [3]. To this end, we performed gene intervention (overexpression or silencing) with MACC1 in colon cancer cell lines. From the RT-qPCR and western blot results, the administration of overexpression MACC1 resulted in a significant increase in MACC1 and c-MET expression, while the administration of silence achieved the exact opposite (*P* < 0.01, Figures 3(d) and 3(e)). Furthermore, not only did overexpressed MACC1 promote the expression of c-MET, but also the expression of c-MET was promoted after the administration of HGF. Meanwhile, the administration of overexpressed MACC1 and HGF stimulated a more pronounced increase in c-MET expression (*P* < 0.01, Figure 3(f)).

### 3.4. Migration Effect of c-MET on Colon Cancer Cells

**Figure 4:**
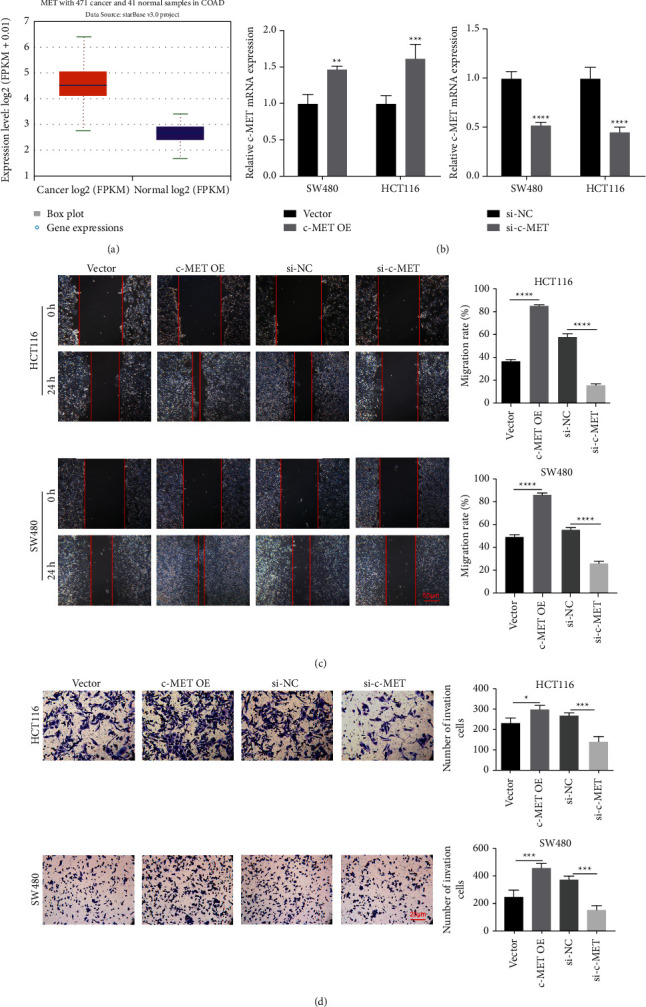
Effect of C-MET on cell migration. c-MET bioletter analysis. (b) qPCR validated the c-MET overexpression and interference efficiency. (c) Scratch test after overexpression and interference of c-MET to detect cell migration. (d) Detection of cell migration by postinvasion assay after overexpression and interference with c-MET. The results are shown as means ± SEM; ^*∗*^*P* < 0.05, ^*∗∗*^*P* < 0.01, and ^*∗∗*^*P* < 0.001.

When we searched the database, we found a correlation between c-MET and cancer migration and invasion (*P* < 0.01, Figure 4(a)). The effect of si-c-MET and c-MET overexpression was first tested to demonstrate this finding (*P* < 0.01, Figure 4(b)). In addition, in order to assess the regulatory role of c-MET in the migration and metastasis of colon cancer cells, we performed a wound surface test and an infiltration test. Wound healing results showed that c-MET overexpression accelerated the migration rate of colon cancer cells (*P* < 0.01), while si-c-MET slowed the migration rate (*P* < 0.01, Figure 4(c)). Transwell experiments showed that c-MET was a metastatic factor in colon cancer because of the overexpression of c-MET leading to an increase invasion of colon cancer cells, while si-c-MET inhibited the invasion of colon cancer cells, which is with the opposite results (*P* < 0.01, Figure 4(d)).

### 3.5. Proliferative Effect of C-MET on Colon Cancer Cells

**Figure 5:**
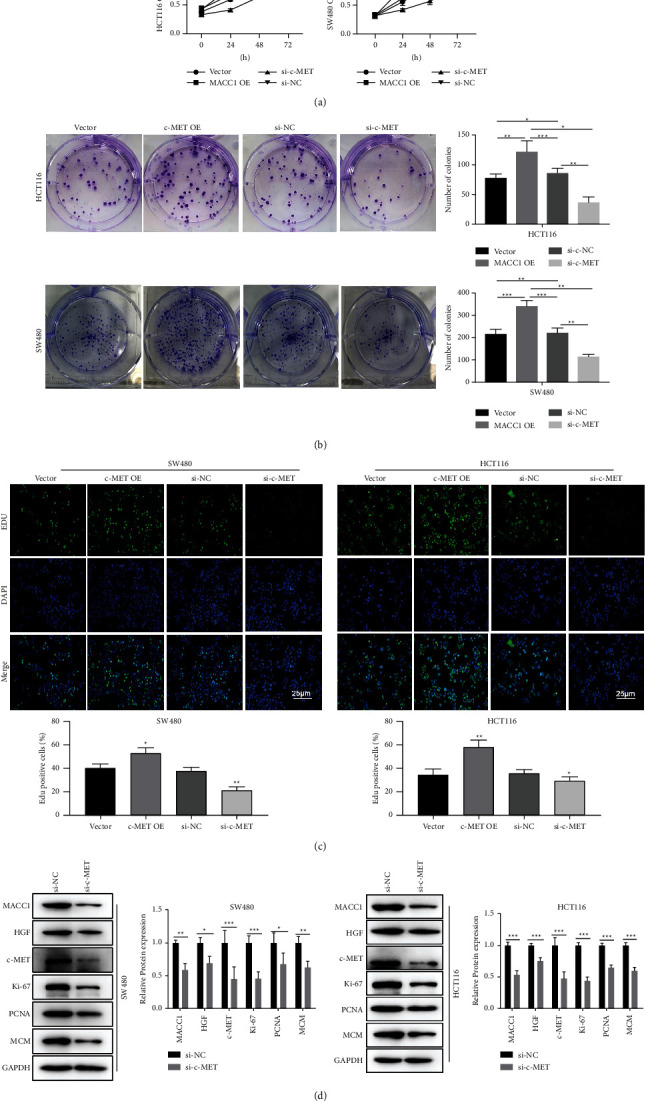
Effect of C-MET on cell migration. (a) Cell viability was determined by CCK-8 assay after overexpression of MACC1 and interference with c-MET. (b) Clone formation occurred after overexpression of MACC1 and interference with c-MET. (c) The cell proliferation was detected by EdU after overexpression and interference of c-MET. (d) The expression of proliferation-related proteins after interfering with c-MET was detected by western blot. The results are shown as means ± SEM; ^*∗*^*P* < 0.05, ^*∗∗*^*P* < 0.01, and ^*∗∗*^*P* < 0.001.

After obtaining the conclusion that c-MET caused an increase invasion of colon cancer cells, we conducted cell proliferation experiments of c-MET on HCT116 and SW480 cell lines. The results of the MTT assay showed that c-MET overexpression significantly increased the proliferation of the colon cells, while si-c-MET had the opposite effect. Clone formation experiment yielded the same results (*P* < 0.01, Figures 5(a) and 5(b)). In an *in vitro* EdU assay, c-MET overexpression increased the proliferation rate of colon cancer cells, while si-c-MET suppressed the colon cancer cell proliferation (*P* < 0.01, Figure 5(c)). Finally, we detected the expression of the proliferation and EMT-related proteins, including Ki-67, PCNA, E-cadherin, vimentin, N-cadherin, and MCM after the knockdown of the c-MET gene using western blot, and the results showed that c-MET knockdown inhibited the ability of proliferation and EMT of colon cancer cells (*P* < 0.01, Figure 5(d)).

### 3.6. MACC1 Accelerates Tumor Growth in the *In Vivo* Model

**Figure 6:**
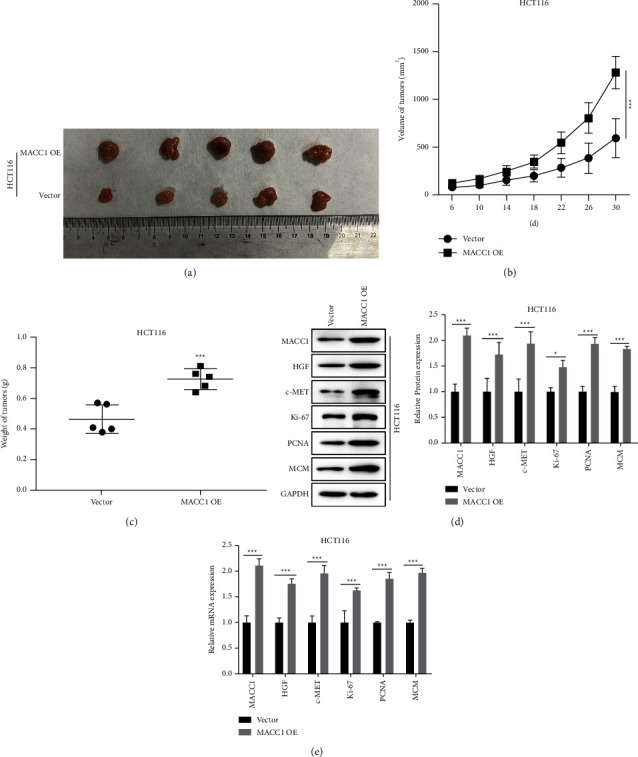
The effect of c-MET on colon tumor growth. (a) Image of tumor tissues. (b) Tumor volumes in the vector group and MACC1 OE group were compared. (c) Tumor weight in the vector group and MACC1 OE group was compared. (d) Western blot assessed the expression levels and statistics of the relevant proteins in tumor tissues relative to control and MACC1 interference groups isolated from nude mice. The results showed as means ± SEM; ^*∗*^*P* < 0.05, ^*∗∗*^*P* < 0.01, and ^*∗∗*^*P* < 0.001.

To further study the effect of MACC1 on tumorigenesis, 10 nude mice were injection transfected with a colon cancer cell line (5 vector and 5 MACC1 overexpression). Thirty days after inoculation, tumors were collected to measure volume and weight. Compared to the tumor tissues in the MACC1 OE group, the tumors in the vector group had a significantly smaller size (*P* < 0.01, Figure 6(a)). Tumor volume was monitored every four days, and tumor tissue in the MACC1 OE group grew much faster than that in the vector group (*P* < 0.01, Figure 6(b)). Tumors in the vector group had significantly lower weights compared to the MACC1 OE group (*P* < 0.01, Figure 6(c)). We examined the expression levels of proliferation and migration-related proteins in the tumor tissues between the vector group and the MACC1 OE group by western blot. The proteins that promote the proliferation and migration of cancer cells in the MACC1 OE cancer tissues were significantly higher than those in the vector group (*P* < 0.01, Figure 6(d)). We also measured mRNA expression in the tumor tissue, and we found that mRNA results were consistent with protein expression (*P* < 0.01, Figure 6(e))

### 3.7. MACC1 Negatively Correlates with Immune Infiltration and Response to ICIs

**Figure 7:**
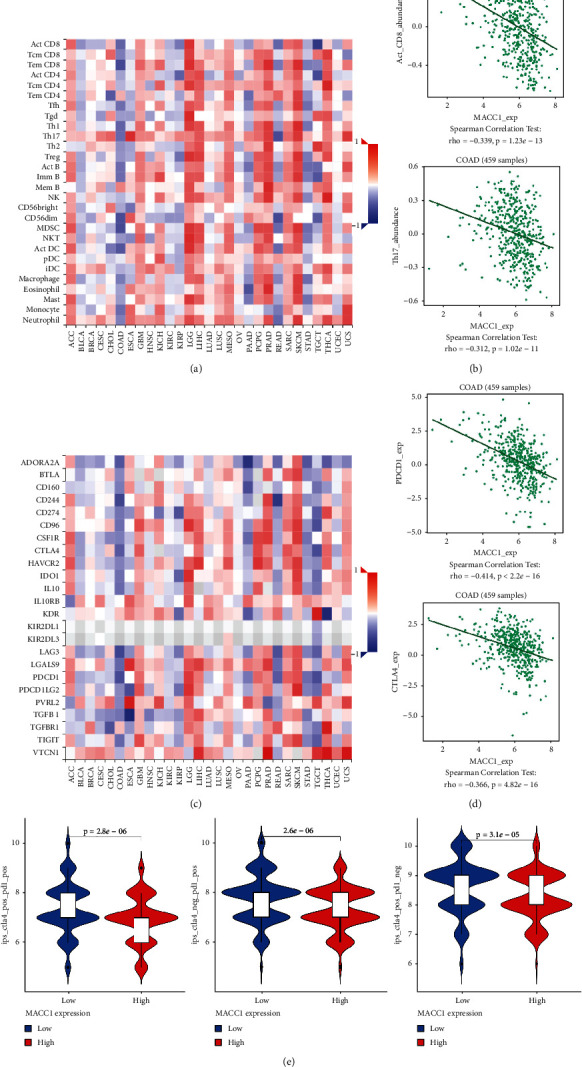
Correlation analysis between MACC1 expression and immune infiltration and immune checkpoint blockage response. (a) The correlation between MACC1 and tumor-associated immune cells was analyzed by TISIDB. (b) The correlation between MACC1 and active CD8+ T cell and Th17 cell infiltration in COAD. (c) The correlation between MACC1 and immune checkpoint biomarkers. (d) The correlation between MACC1 and PDCD1 and CTLA4 expression in COAD. (e) The correlation between MACC1 expression and ICIs response in COAD by TCIA.

Finally, the expression level of MACC1 was negatively correlated with the infiltration level of tumor-infiltrating immune cells (TIICs), which included active, effector memory (TEM), central memory CD8+ T cells, CD4+ T cells, Th1, Th17 cells, and NK cells (Figures 7(a)–7(b)). Moreover, MACC1 expression level also negatively correlates with several immune checkpoint biomarkers, such as CD274, PDCD1, and CTLA4 (Figures 7(c) and 7(d)). Interestingly, high MACC1 expression has a lower response rate with ICIs in COAD, including CTLA-4 combined with PD-1 blockage, PD-1 blockage, or CTLA-4 blockage alone.

## 4. Discussion

Colon cancer is a common tumor of the digestive system. Due to changes in lifestyles, its morbidity has seen a significant increase worldwide. The risk factors for colon cancer include chronic ulcerative colitis, Crohn's disease, colon polyps, and obesity. The early symptoms of colon cancer are often insidious. Therefore, most patients are already in the middle and late stages when they are diagnosed with colon cancer. Recurrence and metastasis are primary factors leading to poor survival and prognosis, as well as an important cause of mortality in colon cancer patients. Therefore, it is particularly important to explore the pathogenesis of colon cancer and find new strategies for early diagnosis and new therapeutic targets.

Tumor invasion and metastasis is a complex and dynamic process, which involves biological behaviors such as the shedding of tumor cells, angiogenesis and metastasis, and formation of new tumors. MACC1 mainly regulates the signal transduction function of hepatocyte conduction factors and their receptor (HGF/c-MET) [7, 27]. Its overexpression can cause the overexpressing of c-MET, thereby triggering the autophosphorylation of various proteins in the cell epithelium and diminishing the adhesion between cells. Meanwhile, it can induce the overexpression of vascular endothelial growth factor (VEGF), which promotes angiogenesis and makes cells with reduced adhesion prone to metastasis [28]. When this effect occurs in tumor tissues, it can accelerate the invasion and metastasis of tumor cells.

We tested NCM460, SW480, HCT116, SW620, and HT-29 cell lines that are commonly used in colon cancer research in this experiment, and the results demonstrated that MACC1 in colon cancer cell lines has high expression [29–34]. We also determined the cell viability and proliferative capacity of each group by CCK-8, EdU, and scratch experiments as well as proliferation markers. In this experiment, we planted the cells in these cell lines in mice for tumorigenicity assay. We analyzed the ki-67, PCNA, and MCM in the tumor, which represent the proliferation and invasion of tumor cells by immunohistochemistry. The results indicated that, after the high expression of MACC1, tumor invasion and proliferation were significantly promoted *in vivo* and *in vitro*.

It has been found that MACC1 has an increasing influence on many cancers. For example, High expression of MACC1 in liver cancer [35], gastric cancer [36], cervical cancer [37], breast cancer [38], non-small-cell lung cancer [39], and so on [40] are related to proliferation, migration, and prognosis. Studies have shown that the expression of MACC1 is changed in various tumors, especially in colon cancer, gastric cancer, and liver cancer [41]. It is not difficult to find a close relationship between MACC1 and c-MET as well as HGF in colon cancer by extracting data from StarBase Pan-cancer. The results of this experiment showed that MACC1 could activate the HGF and then stimulate the phosphorylation of c-MET, thus enhancing the invasion ability of tumor cells.

Moreover, the upregulation of the HGF was found to be involved in the mitosis of primary hepatocytes [42] and has the effects of strongly promoting division, inducing epithelial cell migration, invasion, and inducing angiogenesis. c-MET is a specific membrane surface receptor of HGF that mediates the activity of HGF. The HGF/c-MET pathway was observed to be abnormally activated in a variety of tumors, causing overexpression, amplification, and mutation of c-MET gene, ultimately promoting the growth, invasion, and metastasis of tumors. Thus, the development, progression, and metastasis of tumors can be effectively inhibited by blocking the HGF/c-MET pathway. Therefore, the HGF/c-MET signal has been used as a therapeutic target in ovarian cancer clinical trials [43]. This experiment showed that there was also an upstream-downstream relationship between MACC1 and c-MET in colon cancer cells and tissues, both of which formed the MACC1/HGF/c-MET axis [44].

Then, we identified the MACC1/HGF/c-MET axis in colon cancer and tried to explore whether this axis could interfere with colon cancer invasion and metastasis by regulating MACC1 or by blocking the expression of downstream c-MET. According to the results of *in vitro* experiments, the recession of MACC1 and c-MET in colon cancer cells can indeed regulate the proliferation and migration of cancer cells. Specifically, inhibition of the MACC1/HGF/c-MET axis contributes to the treatment and prognosis of colon cancer and also helps to suppress the proliferation and invasion of colon cancer cells.

At last, we also found that MACC1 is correlated with lower immune cell infiltration and immune checkpoint biomarkers in COAD by the TISIDB tool and immunophenoscore analysis. The immune cell infiltration is a critical requirement for the response of ICIs to tumor. Lower immune cell infiltration, especially CD8+ T cell infiltration, will impact the immune response when treatment with ICIs. Our results also identify, for the first time, MACC1 as a possible new biomarker for predicting ICI response in COAD.

## 5. Conclusions

In conclusion, our research results showed that MACC1 has an effect of promoting the development and progression of colorectal cancer *in vitro* and in *vivo*. Furthermore, it was found that MACC1 can inhibit the proliferation and metastasis of colorectal cancer and induce apoptosis by regulating the HGF/c-MET pathway and could be a novel biomarker for predicting ICIs response in colorectal cancer. These results provide new insights into the molecular mechanisms of MACC1 treatment in colorectal cancer. Our findings are expected to ultimately accelerate the development of treatment for colorectal cancer.

## Figures and Tables

**Table 1:** Primer used in this study.

	Forward (5′-3′ sequence)	Reverse (3′-5′ sequence)
qRT-PCR		
MACC1	CATTTTCGGTCAGGAAGAATTGC	TGGAAGCATTATTACCACGAAGG
GAPDH	CATGAGAAGTATGACAACAGCCT	AGTCCTTCCACGATACCAAAGT
c-MET	AATGCTGGCACCCTAAAGC	AAGATCGCTGATATCCGGG
HGF	ACATCGTCACTTCTGGC	ATCCATCCTATGTTTGTTCG
Ki-67	ACAGCCGCCGAACAGACT	GCACATAGGAAACCACCT
PCNA	TGACAAATGCTTGCTGACC	AGGATGGAGCCCTGGACC
MCM7	CCTACCAGCCGATCCAGTCT	CCTCCTGAGCGGTTGGTTT
SiRNA		
Si-c-MET	GAGCCAGCCTGAATGATGA	GGAGCAACGAGGATTACCT
Si-MACC1-2	AAGAGGGGACGGGGACACGGCTT	TTGGCGAACCGGAACAGGGGACG
Si-MACC1-1	GAGTGCTCACTATGGAAATAA	TTATTTCCATAGTGAGCACTC
Si-NC	GTTCTCCGAACGTGTCACGTA	CAAGAGGCTTGCACAGTGCAT

## Data Availability

Data are available from TISIDB (https://cis.hku.hk/TISIDB/); IPSs of TCGA COAD patients were downloaded from The Cancer Immunome Atlas (TCIA).

## Abbreviations

c-MET: Hepatocyte growth factor receptor
HGF: Hepatocyte growth factor
MACC1: Metastasis-associated in colon cancer-1
siRNA: Small interfering RNA
RT-qPCR: Real-time polymerase chain reaction
FBS: Fatal bovine serum
MTT: 3-(4,5-Dimethyl-2-thiazolyl)-2,5-diphenyl-2-H-tetrazolium bromide
TCGA: The Cancer Genome Atlas.
